# Detection of cell markers from single cell RNA-seq with sc2marker

**DOI:** 10.1186/s12859-022-04817-5

**Published:** 2022-07-12

**Authors:** Ronghui Li, Bella Banjanin, Rebekka K. Schneider, Ivan G. Costa

**Affiliations:** 1 Joint Research Center for Computational Biomedicine, Institute for Computational Genomics, RWTH Aachen University, Aachen, Germany; 2 Department of Cell Biology, Institute for Biomedical Engineering, RWTH Aachen University, Aachen, Germany

**Keywords:** Single cell RNA-seq, Marker identification, Maximum margin

## Abstract

**Background:**

Single-cell RNA sequencing (scRNA-seq) allows the detection of rare cell types in complex tissues. The detection of markers for rare cell types is useful for further biological analysis of, for example, flow cytometry and imaging data sets for either physical isolation or spatial characterization of these cells. However, only a few computational approaches consider the problem of selecting specific marker genes from scRNA-seq data.

**Results:**

Here, we propose sc2marker, which is based on the maximum margin index and a database of proteins with antibodies, to select markers for flow cytometry or imaging. We evaluated the performances of sc2marker and competing methods in ranking known markers in scRNA-seq data of immune and stromal cells. The results showed that sc2marker performed better than the competing methods in accuracy, while having a competitive running time.

**Supplementary Information:**

The online version contains supplementary material available at 10.1186/s12859-022-04817-5.

## Background

Advances in single-cell RNA sequencing (scRNA-seq) methods have revolutionized biomedical research by allowing the transcriptomes of millions of cells to be studied at the same time. This has helped to uncover molecular processes that drive cell differentiation and complex diseases [1, 2]. Many computational methods have been proposed for scRNA-seq data analysis [1, 3], including unsupervised analysis to characterize novel and disease-specific cell sub-populations such as rare stromal and immune cells [4–6] that cannot be detected by other methods. The delineation of small panels with marker genes that characterize such sub-populations is of particular importance for further molecular characterization and validation of the detected cells. For example, flow cytometry can be used to physically isolate cells and quantify cell populations or the expression of markers for both research and clinical applications [7]. However, flow cytometry requires a small panel of antibodies (< 50) that target previously characterized cell surface proteins that can be used as markers for cell types of interest. Multiplex immunohistochemistry (IHC) imaging allows protein abundance to be measured at a cellular level in tissue cross-sections, which allows cell identification in a spatial context. IHC can also be performed on small panels of markers (>30) with IHC compatible antibodies [8]. However, there is a lack of computational methods to explore the high gene coverage of scRNA-seq for delineation of cell markers from novel cell subtypes, as detected by cluster analysis of scRNA-seq data, for further delineation with antibody-based flow cytometry or IHC imaging.

To our knowledge, only a few bioinformatics methods explicitly tackle this problem [9], i.e. detection and ranking of few cell specific markers from single cell data. COMET [10], Hypergate [11], CombiROC [12] and RANKCORR [13] are based on finding an optimal threshold value for a particular gene to split cells into two groups, so that true positives and true negatives are maximal and false positives and false negatives are minimal. COMET uses an XL-minimal HyperGeometric (mHG) test to find a threshold that maximizes the enrichment of a cell type given a panel with up to four genes. COMET also has a database with surface marker genes to guide the selection of markers for flow cytometry. A downside of COMET is that it has high execution times and cannot cope with data sets with a high number of cells. Hypergate uses a purity score statistic to find markers in scRNA-seq data that distinguish different cell types, but its current implementation only provides a single marker per cell. RANKCORR explores a non-parametric approach, i.e. ranking gene expression, and using sparse binomial regression to find the optimal set of markers for distinct cells [13]. Finally, CombiROC explores the area under the ROC curve statistics of marker combinations find the best panel combinations [12]. Commonly used scRNA-seq analysis frameworks such as Seurat [3] and MAST [14] provide parametric models that can also be used for marker detection. However, these methods are known to select markers with low cell specificity; i.e., highly ranked marker genes are highly expressed in the target cell, but may also be expressed in other cell types.

Here, we propose sc2marker, which uses a non-parametric feature selection method based on maximum margin to search for marker genes in clustered scRNA-seq data. sc2marker considers the distance of true positive and true negative cells to the optimal threshold (maximum margin) to score the best marker genes. Competing methods (COMET, Hypergate, CombiROC and RANKCORR) do not use the distance of cells to the classification threshold to rank marker genes. sc2marker has databases that contain markers with antibodies tailored for particular applications, including IHC (11,488 protein markers) and IHC staining (6176 protein markers), extracted from the Human Protein Atlas [8]. We also build a database that contains proteins with antibodies for flow cytometry (1357 protein markers), which were categorized as cell surface or extracellular matrix proteins in either the Cell Surface Protein Atlas [15], OmmiPath [16], CellChatDB [17], or the HUGO database [18]. These databases contain human proteins and antibodies, which have been more broadly validated than those in other organisms. sc2marker also provides similar databases that have been tailored for mouse by combing both a small set of antibodies validated in mouse or proteins that share high sequence similarity between mouse and human [19–21]. These databases support the feature selection task because feature selection can be restricted to the gene spaces related to these proteins. Regarding the competing methods, only COMET has an antibody database, but it is restricted to flow cytometry markers.

sc2marker is implemented in R and compatible with Seurat [3] objects. The results are presented as an intuitive graphical representation with plots and interactive tables. The tables contain a ranking of all the selected markers and include relevant information such as the source of the database, links to vendors, and links to the antibody registry [22]. We evaluate the capability of sc2marker and competing methods to detect known flow cytometry and imaging markers of well characterized immune cells [10]. For this, we used five publicly available scRNA-seq data sets of immune cells and evaluated the performances of these methods in ranking known marker genes. We also used sc2marker with two scRNA-seq datasets of mouse bone marrow stromal cells reported previously [4, 5], and evaluated its performance in previously validated markers of poorly characterized and similar mesenchymal stromal cell populations.

## Methods

### sc2marker

**Fig. 1:**
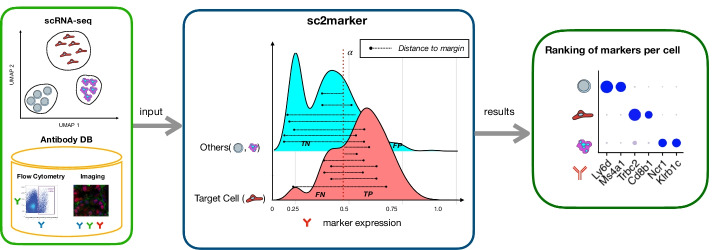
Schematic representation of the sc2marker framework: The input is a clustered single-cell RNA-seq data set and a list of antibodies for the selected application; i.e., flow cytometry or imaging. For every potential marker and cell type, sc2marker finds an optimal threshold $\alpha$ (or margin) with maximal distances to true positives (TP) and true negatives (TN) and low distances to false positives (FP) and false negatives (FN). The threshold score is used to rank markers for each cell type

We developed sc2marker to predict markers for particular cell types. sc2marker uses a normalized gene-by-cell matrix as input (*X*) and cell labels obtained by clustering the cells (Fig. 1). Then criteria based on maximum margin are used to find optimal thresholds and rank genes according to their power in distinguishing a target cell type from other cells in a data set. Moreover, sc2marker can restrict the feature space to consider only genes that have validated antibodies for a particular down-stream application: flow cytometry, IHC or immunocytochemistry (ICC) imaging, or a user provided human or mouse database. sc2marker outputs a list of markers and its respective visual representation for each cell type.

### Feature selection using a maximum margin model

Let $\mathbf {X} \in \mathbb {R}^{n x m}$ represent the cell-by-gene matrix, where *n* is the number of cells and *m* is the number of genes. All genes are brought to a similar scale as follows:

1 $$\begin{aligned} \hat{x}_{ij} = {{x_{ij} - min(x_{j})}\over {max(x_{j})-min(x_{j})} } \end{aligned}$$

where $x_{ij}$ is the expression of gene *j* in cell *i*, and $x_{j}$ is a vector that represents the expression of gene *j* for all cells.

For a given cell type *p*, a class vector $y^p$ is defined as follows:

2 $$_{i}^{p} = \left\{ {\begin{array}{*{20}l} {1,} \hfill & {{\text{if cell}}\;i\;{\text{is}}\;{\text{cell}}\;{\text{type}}\;p.} \hfill \\ { - 1,} \hfill & {{\text{otherwise}}.} \hfill \\ \end{array} } \right.$$

sc2marker uses a univariate maximum margin function to find the best threshold for a given gene *j* as follows:

$$\begin{aligned} \begin{aligned} \alpha _j = \underset{\alpha _j}{argmax}\left( \sum _i \left( y^p_i (x_{ij} - \alpha _j)\right) \right) \end{aligned} \end{aligned}$$

where $\alpha _j \in (0,1)$ is the optimal cutoff to classify gene *j* as cell type *p*.

The class label $y^p$ is typically highly imbalanced; i.e., the number of cells for a given cell type (positives) is usually smaller than the number of other cell types (negatives). Also, sparsity of single cell sequencing data, i.e. no expression might be detected for lowly expressed genes, asks for a milder penalization of false negative events. Therefore, we adapted the previous univariate maximum margin function to consider the distances of the true predictions, such that the distances to the true positive predictions have a higher weight than the distances to the true negative predictions, that is:

3 $$\begin{aligned} \alpha _j = \underset{\alpha _j}{argmax}(\mathbf {GeneSplit}(\alpha _{j})), \end{aligned}$$

where

4 $${\mathbf{GeneSplit}}(\alpha _{j} )\quad = |D_{j} |\sum\limits_{{x_{{ij}} \in A_{j} }} {y_{i}^{p} } (x_{{ij}} - \alpha _{j} ) + |A_{j} |\sum\limits_{{x_{{ij}} \in D_{j} }} {y_{i}^{p} } (x_{{ij}} - \alpha _{j} ) + |B_{j} |\sum\limits_{{x_{{ij}} \in C_{j} }} {y_{i}^{p} } (x_{{ij}} - \alpha _{j} )$$

where $A_{j}$ is equal to the set of true positives $\{ \hat{X}_{ij}| y^p_i=1, \hat{X}_{ij} > \alpha \}$, and $B_{j}$ is the set of false negatives $\{ \hat{X}_{ij}| y^p_i=1, \hat{X}_{ij} {\le } \alpha \}$. The set of true negatives $D_{j}$ and false positives $C_j$ is defined accordingly. Next, sc2marker performs a grid search to find the optimal $\alpha$ for each gene *i* and cell type *p*. By default, sc2marker evaluates values from 0 to 1 in increments of 0.01. Of note, the previous equation omits the term associated with false negative observations (*B*), as these might arise from single cell data sparsity.

Finally, all genes for a given cell type *p* with optimal $\alpha$ are ranked using the following criteria:

5 $$\begin{aligned} Gene.ranking.score(a_j) = GeneSplit(a_j) \cdot TPR \cdot TNR \cdot FC^2 \end{aligned}$$

where *TPR* is the true positive rate $\left( \frac{|A_{i}|}{|A_{i} \bigcup B_{i}|}\right)$ and *TNR* is the true negative rate $\frac{|D_{i}|}{|D_{i} \bigcup C_{i}|}$ and *FC* is the log fold change of the gene expression of the positive and negative predictions $\frac{mean(A_i \bigcup B_i) + \sigma }{mean(C_i \bigcup D_i) + \sigma }$, where $\sigma$ is a pseudo count (0.01 as default). The *Gene*.*ranking*.*score* reinforces the importance of true positive and true negative predictions for marker ranking. The fold change (FC) guarantees a high difference in the expression levels of the marker in the two groups. The previous equation detects positive markers; i.e., those with higher expression in the cell type of interest. Negative markers are estimated by inverting the expression values.

To filter low-quality candidate markers, sc2marker ignores genes whose expression is detected in less than 15% (by default) of the cells in a cluster of choice (positives). It also ignores markers with true negatives lower than 0.65 (default value).

#### Database of antibodies

Another important feature of *sc2marker* is the database that contains known available antibodies. We collected genes that encode proteins with validated antibodies that have been used in different kinds of experiments including IHC and ICC from the Human Protein Atlas [8]. For flow cytometry, we catalogued antibodies indicated for flow from commercial manufacturers. We also collected genes annotated as being clusters of differentiation genes (HUGO [18]), cell surface genes (Cell Surface Protein Atlas [15]), and extracellular matrix genes (OmniPath [16], CellchatDB [17]). Proteins from OmniPath and the Cell Surface Protein Atlas whose function was computationally predicted were not included. Finally, only genes that encode proteins with validated antibodies in at least one of these databases were considered as flow cytometry markers.

All previous antibody databases have focused on human proteins. To construct a mouse version of these databases for imaging data, we selected the small number of proteins in the Human Protein Atlas that have also been validated in a mouse brain cell line (979 genes for IHC and ICC). To expand the number of proteins, we used a strategy that is commonly reported in the literature; i.e., we considered only proteins with high sequence conservation in the antigen regions between mouse and human (90$\%$) [19–21, 23]. Using this strategy, we obtained 7306 and 6477 proteins that had associated IHC and ICC data, respectively. For the flow cytometry data, there are only a few antibodies that have been reported by vendors to be validated for mouse. To expand the number of antibodies, we again considered proteins with high sequence conservation between mouse and human (90$\%$) and constructed a flow cytometry mouse database that contained 528 proteins. Details of the antibody databases used by sc2marker are provided in Table 1.

**Table 1:** Antibody databases used by sc2marker

Category	Source	Number
ICC	Human Protein Atlas	12813
IHC	Human Protein Atlas	15320
Flow	Antibody vendors, Cell Surface Protein Atlas, HUGO, OmniPath, CellchatDB	1357
ICC_Mouse	Human Protein Atlas (antigen conservation or validated in mice )	6477
IHC_Mouse	Human Protein Atlas (antigen conservation or validated in mice)	7306
Flow_Mouse	Antibody vendors, Cell Surface Protein Atlas, HUGO (antigen conservation or validated in mice)	528

*ICC* immunocytochemistry, *IHC* immunohistochemistry, *Flow* flow cytometry

In all cases, we used the gene symbol as the protein identifier. The appropriate database is used by sc2marker to limit the gene search space. Antibody information and a link to the antibody register [22] are given in the sc2marker output to provide users with the appropriate information for final marker selection. sc2marker also allows a user to provide their own marker database, which can be easily input as a csv file. Users can execute sc2marker with any database combination. sc2marker provides the database and annotation details for a given marker, which allows users to trace the evidence (validated, homology, or user provided) that supports the marker.

### Datasets

We used publicly available scRNA-seq data sets to benchmark sc2marker and compare it with competing methods. In particular, we explored data sets of immune cells for which flow cytometry markers are known to be well characterized for cell types.

#### Mouse Cell Atlas scRNA-seq data for spleen and lung

The Mouse Cell Atlas (MCA) [2] is a database that contains more than 400,000 single-cell transcriptomics profiles from 51 mouse tissues, organs, and cell cultures constructed by Microwell-seq. Cell types in the MCA data have been fully defined by manual annotation and cover more than 800 major cell types and more than 1000 cell subtypes. We obtained scRNA-seq data from mouse spleen and lung as a cell-by-gene matrix that was normalized using the LogNormalize function in the Seurat package) [3] with scale.factor = 10,000 and scaled using the ScaleData function with default settings. Principal component analysis was performed on the scaled matrix and the first 30 principal components were used to run a UMAP dimension reduction. The spleen data set (MCA-spleen) contained 1970 cells that were characterized in 10 distinct cell types (erythroblast, plasma cell, neutrophil, T cell, marginal zone B cell, monocyte, dendritic cell, macrophage, granulocyte, and natural killer (NK) cell). The lung data set (MCA-lung) contained 6940 cells that were characterized in 31 distinct cell types, including B cell, T cell, and NK cells.

**Fig. 2:**
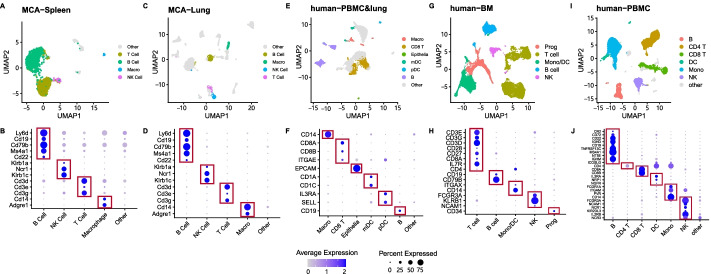
UMAP dimension reduction and dotplots of gene expression of markers used as true labels. Five cell types and five data sets were used. Mouse Cell Atlas (MCA)-Spleen (**A, B**), MCA-Lung (**C, D**), Human-Lung (**E, F**), Human-BM (bone marrow) (**G, H**), and Human-PBMC (human peripheral blood mononuclear cells) (**I, J**). In the dotplots, the size of the dot indicates the number of cells expressing the gene and the color intensity indicates the expression level

We used known cell markers for the well characterized immune cells (B cell, T cell, NK cell, and macrophages) as true class labels in both the MCA-spleen and MCA-lung data sets [10] (Table 2). The UMAP dimension reduction for the immune cell types and gene expression of true labels are shown in Fig. 2.

**Table 2:** Known flow cytometry cell markers for major immune cells

Cell type	Markers seta
B cell	Ly6d, Cd19, Ms4a1, Cd22, Cd79b
T cell	Cd3d, Cd3e, Cd3g
NK cell	Ncr1, Klrb1a, Klrb1c
Macrophages	Cd14, Adgre1

#### Human multiome of peripheral blood mononuclear cells

We used a multiome data set (CITE-seq with RNA and protein) of human peripheral blood mononuclear cells (human-PBMC) that contains 16,1764 human white blood cells with scRNA-seq data of 20,729 genes and 228 surface proteins obtained from  https://atlas.fredhutch.org/nygc/multimodal-pbmc/. Because both protein and RNA data were available, we performed an independent selection of true labels on the protein data using the standard Seurat protocol. We used the selected true labels to evaluate the marker detection methods that consider only scRNA-seq data.

Pre-processing and dimensional reduction were performed independently on both the RNA and protein data. Weighted nearest neighbor (WNN) analysis [3] was used to calculate the closest neighbors of each cell in the data set based on a weighted combination of RNA and protein similarities. UMAP dimension reduction and clustering were performed on the WNN graph. Eight cell types were identified: monocytes, B cells, NK cells, CD4+ T cells, CD8+ T cells, other T cells, dendritic cells, and others. Then, we performed a differential abundance analysis of the protein data using Seurat (Wilcoxon rank sum test) to derive cell-specific markers (true labels). This analysis and cell annotation were obtained by following the Seurat 4 tutorial, and therefore serve as an independent method for delineation of true labels [3] (see https://satijalab.org/seurat/articles/multimodal_vignette.html).

We obtained the following true label markers: CD21, CD72, CD22, IgD, CD19, CD268, CD20, CD73, CD275, and IgM (CR2, CD72, CD22, IGHD, CD19, TNFRSF13C, MS4A1, NT5E, ICOSLG, and IGHM) for B cells; CD123, CD304, and CD271 (IL3RA, NRP1, and NGFR) for dendritic cells ; CD8 (CD8A, CD8B) for CD8+ T cells; CD4 for CD4+ T cells; CD64, CD11b, CD155, CD14 (FCGR1A, ITGAM, PVR, CD14) for monocytes; and CD16, CD56, CD335, CD122, CD337, and CD158B (FCGR3A, NCAM1, NCR1, KIR2DL3, IL2RB, and NCR3) for NK cells.

#### Human multiome of human bone marrow

We used a human bone marrow CITE-seq data set (human-BM) that contains 30,672 human bone marrow cells with 17,009 genes and 25 surface proteins [24]. Five major cell types were identified: T cells, B cells, progenitor cells, NK cells, and monocyte/ dendritic cells. We use the same strategy as for the human-PBMC data for pre-processing and delineation of cell-specific markers (true labels). We obtained the following true label markers: CD3C, CD3G, CD3D, CD27, CD28, IL7R, CD8A, and CD4 for T cells; CD19 and CD79B for B cells; CD34 for progenitor cells; ITGAX and NCAM1 for monocyte/dendritic cells; and FCGR3A, KLRB1, and NCAM1 for NK cells.

#### Human multiome of PBMC and lung cells

We also used data from a multiome protocol with PBMC and lung cells (human-PBMC&lung) that contained 10,470 cells (7108 lung, 3362 PBMC) with 33,514 genes and 52 surface proteins  [25] obtained from the  https://archive.softwareheritage.org/browse/revision/1c7fcabb18a1971dc4d6e29bc3ed4f6f36b2361f/link. Nine major cell types were identified: CD4+ T cells, CD8+ T cells, B cells, plasmacytoid dendritic cells, NK cells, myeloid dendritic cells, epithelial cells, macrophages, and monocytes. We used the same strategy as for the human-PBMC data for pre-processing and delineation of cell-specific markers (true labels). The following true label markers were obtained: CD8A, CD8B, and ITGAE for CD8+ T cells; CD19 for B cells; CD1A and CD1C for myeloid dendritic cells; IL3RA and SELL for plasmacytoid dendritic cells; and EPCAM for epithelial cells.

#### Mouse bone marrow stromal cells

We used a mouse bone marrow stromal cells data set measured by droplet-based scRNA-seq that contained common bone marrow cell types and rare cells [5]. The data set contained 7497 cells and 16,701 genes that were grouped in 32 cell types, including mesenchymal, immune, neuronal, endothelial, and hematopoietic progenitors cells. We used the pre-processed data as provided by Baccin and colleagues [5]. We focused on the characterization of the mesenchymal cell types. Because of the lack of known markers for these cell types, we did not used these data for benchmarking, but used the data in a exploratory analysis with sc2marker.

#### Myelofibrosis mouse bone marrow stromal cells

We used a single cell sequencing data with stromal cells from a myelofibrosis mouse model previously described in [4]. In short, we obtained an pre-processed data with 2294 cells and eight clusters including from https://doi.org/10.5281/zenodo.3979087. We focus on the characterization of mesenchymal stromal cells (MSC), osteolineage cells, adventitial fibroblasts and schwann cell progenitors. We have focused on the detection of markers of two MSC

### Competing methods

We evaluated the performances of competing methods COMET [10], Hypergraph [11], RANKCORR [13] and CombiROC [12], as well as statistical tests used for selection of cluster-specific markers in scRNA-seq data, including the student t-test, Wilcoxon rank sum test, logistic regression (all implemented in Seurat [3]), and MAST [14]. The evaluations also included base line methods such as expression fold change (FC) and receiver operating characteristic (ROC)-based feature selection [26].

#### Seurat marker detection

We evaluated the statistical methods provided by Seurat [3] to compare the expression distribution of a gene between two groups of cells; i.e. cells of a target cell type *p* and other cell types. These methods include the student *t*-test, Wilcoxon rank sum test, and logistic regression implemented in the Seurat package. We used the provided statistics (*p*-values) to rank the candidate marker genes, and explored the simple use of expression FC as provided by Seurat.

#### MAST hurdle model

MAST [14] explores a hurdle model tailored to scRNA-seq data that assumes the log of expression follows a normal distribution to identify genes that are differentially expressed between two groups of cells. The fraction of genes that are detected in each cell is used as a covariate to fit a logistic regression model. This process helps decrease background correlation between genes. We used the *p*-value to rank the candidate genes returned by MAST.

#### Receiver operating characteristic (ROC) curve

For a given cell type *p*, ROC takes a single gene as the classifier. For gene *j*, the ROC curve goes through $\alpha _j \in (min(x_{j}),max(x_{j}))$. The set of true positives is equal to $\{\hat{X}_{ij}| y^p_i=1, \hat{X}_{ij} > \alpha _j \}$ and the set of false negatives is $\{ \hat{X}_{ij}| y^p_i=1, \hat{X}_{ij} \le \alpha \}$. True negatives and false positives are defined accordingly. The ROC curve of gene *j* is built based on the true positive rate (TPR) and true negative rate (TNR) of different $\alpha _j$ as follows:

6 $$TPR = \frac{{TruePositive}}{{TruePositive + FalseNegative}}$$

7 $$TNR = \frac{{TrueNegative}}{{TrueNegative + FalsePositive}}.$$

The area under the (ROC) curve (AUC) is calculated and the classification power is calculated as $(abs(AUC-0.5)) * 2)$. Classification power values range from 0 to 1, where 1 indicates perfect classification. This score is used to rank the predictions.

#### COMET

COMET [10] is a framework for combinatorial prediction of single-gene or multi-gene marker panels. COMET performs non-parametric exhaustive searches to predict marker panels for all combinations for up to four markers. COMET implements a XL-mHG test to calculate the best split value of two clusters and ranks the features based on the XL-mHG *p*-value and then the log2 FC of mean expression within and outside the cluster of interest. For a given cell type *p*, the mHG test first ranks the class vector $y^p$ by the gene expression in decreasing order, and then calculates the mHG test *P*value. The XL-mHG test uses two additional parameters X and L, where X indicates the minimum true positive rate (TPR) and L indicates the maximum of predicted positive cases. COMET has a list of 796 surface marker genes; 13 are obsolete gene symbols. Most of these genes (661) are common with the sc2marker flow database (135 genes are unique to COMET and 696 are unique to sc2marker). We evaluated COMET with this gene list (COMET+DB) and without a gene list (COMET). sc2marker also allows users to explore the COMET database using the option “category=FlowComet”.

#### Hypergate

Hypergate [11] uses a non-parametric score that explores true positives (TP), false positives (FP), false negatives (FN) and true negatives (FN) (as defined above) in a measure that combines both purity (sensitivity) and yield (specificity). The Hypergate F1 score is calculated as follows:

8 $$\begin{aligned}&F1 = 2 \cdot \frac{Precision \cdot Recall}{ Precision + Recall} \end{aligned}$$

9 $$\begin{aligned}&Precision = \frac{\#TP}{\#TP + \#FP}, Recall = \frac{\#TP}{\#TP + \#FN} \end{aligned}$$

where *TP* is the set of true positives $\{\hat{X}_{ij}| y^p_i=1, \hat{X}_{ij} > \alpha \}$, and *FP* is the set of false positives $\{ \hat{X}_{ij}| y^p_i=1, \hat{X}_{ij} \le \alpha \}$. The set of false negatives (*FN*) is defined accordingly as *TP* and *FP*. Hypergate implementation only detects up to four markers per cell type; therefore, we reimplemented the Hypergate criteria within our tool to rank all markers as do all other evaluated methods.

#### CombiROC

CombiROC [27] explores ROC curves from a combination of predictors (between 1 and 5) ranked by specificity, sensitivity and area under the ROC curve (AUC). It was executed with default parameters. Of note, due to computational demands, we were only able to execute CombiROC after filtering for genes in the antibody database.

### RANKCORR

RANKCORR [13] works by creating ranks of mRNA count data and then searches for a hyperplane that linearly separates a small number of marker genes (between 4 and 8). Moreover, RANKCORR supports multi-class marker selection. For comparative purposes, we set RANKCORR for the two-class classification problem (target cells vs. other cells). Otherwise, we used default parameters.

## Results

### Benchmarking of methods for cell marker detection for flow cytometry data

We used five distinct data sets (MCA-lung, MCA-spleen, human-PBMC, human-BM, and human-lung &PBMC) to evaluate the predictive performance of sc2marker and competing methods (t-test, Wilcoxon rank sum test, MAST, linear regression, ROC, FC, COMET, Hypergate, CombiROC and RANKCORR) for recovery of cell-specific marker genes. We evaluated all methods by either considering all genes or only genes in sc2marker antibody DB. Due to high computational time, we could only evaluate CombiROC with the antibody DB. Their performances with the MCA-lung and MCA-spleen data were evaluated by ranking true marker genes, which are a collection of well-known flow markers for immune cells, as was proposed for COMET [10] (see Table 2). For the human data sets (PBMC, BM, and Lung &PBMC), we define true labels from an independent analysis of the matching protein data using the standard Seurat pipeline for CITE-seq data. Marker predictions were performed by providing only scRNA-seq data. Visualization of the scRNA-seq clusters and expression values of the markers (true labels) are shown in Fig. 2.

**Fig. 3:**
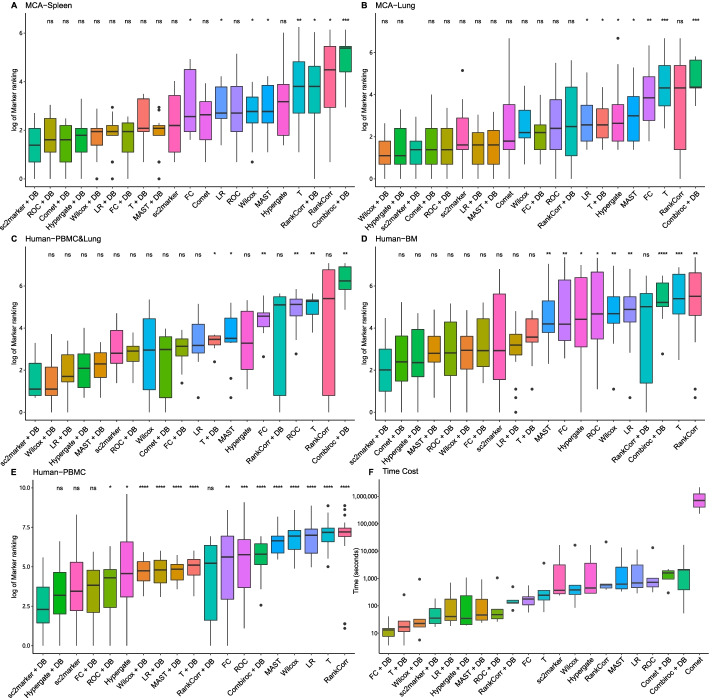
Ranking of markers and computational time for all the evaluated methods: Distribution of the ranks of true markers for the MCA-Spleen (**A**), MCA-Lung (**B**), Human-Lung &PBMC (**C**), Human-BM (bone marrow CITE-seq data) (**D**), and Human-PBMC (human peripheral blood mononuclear cells CITE-seq data) (**E**) data sets for all evaluated methods. For simplicity, the distributions and ranks of all the cell types were combined for a given method and data set. The methods that gave lower ranks for the true markers were the best in recovering the true cell markers. (**F**) Distribution of computational time for all the evaluated methods in seconds. Methods are ranked by increasing median value and values are shown in log 10 scale. The statistical significance of the best overall method (sc2marker + DB) compared with the other computing method is indicated as; * p-value < 0.1, ** p-value < 0.01, *** p-value < 0.001, **** p-value < 0.0001 by the Wilcoxon rank sum test (adjusted using the Bonferroni correction)

The distributions of ranks of all true labels for each method and data set are shown in Fig. 3A–E. Marker predictions were performed for each cell type independently, but cell type-specific ranks were combined for simplicity. A good performing method should have low rank scores; i.e., true marker genes are ranked as 1, 2, 3, and so on. sc2marker+DB had the lowest rank distribution in four out of five data sets and was third in another data set. Another good performing tool was Hypergate+DB, which was among the four top performing methods in all data sets. Among the methods that do not use an antibody database, sc2marker had the lowest rank scores in all data sets. The statistical methods implemented in Seurat (*t*-test and linear regression) had poor performances in most data sets. The complete results with ranking of all the methods for each cell type and data set are provided in Additional files 1, 2, 3, 4, 5 and 6.

The computational times for most of the methods were less than one hour for the MCA-lung and MCA-spleen data sets (Table 3). An exception was COMET, which required 64 hours for the MCA-spleen and 24 days for the MCA-lung data set and failed to execute in the large human-PBMC data set because of the large number of cells. CombiROC failed to run without the antibody DB in all data sets. The use of the antibody DB reduced the computational time for all methods by at least 10-fold (Fig. 3F; Table 3), with the exception of RANKCORR, which had a decrease of five fold. Indeed, sc2marker+DB was among the top 4 fastest methods only being outperformed by the statistical approaches FC, *t*-test and Wilcoxon test (Fig. 3F). The maximum computational time of sc2marker+DB was 3 minutes to detect markers in the large human-PBMC data set. This reinforces the idea that using an antibody database improves both ranking accuracy and computational time for sc2marker and all evaluated methods.

**Table 3:** Execution time of methods in seconds. T, student *t*-test; LR, linear regression; ROC, receiver operating characteristic; FC, fold change

Method	MCA-Spleen	MCA-Lung	Human-Lung &PBMC	Human-BM	Human-PBMC
COMET	233,760.604	2,143,037.250	Failed	Failed	Failed
COMET + DB	297.737	1,837.851	260.130	383.059	Failed
CombiROC + DB	53.112	385.255	2130.589	2080.815	16854.740
MAST	335.188	2,584.087	418.121	615.921	13,457.332
MAST + DB	24.285	173.521	29.920	46.128	918.817
Wilcoxon	84.161	563.938	260.130	383.059	16,507.774
Wilcoxon + DB	7.623	32.223	43.817	24.566	938.315
T	59.072	367.252	162.578	243.478	3,634.189
T + DB	7.555	27.483	17.432	16.956	253.804
LR	285.929	3,107.313	464.449	684.169	11,372.861
LR + DB	18.368	174.837	29.061	40.596	701.804
ROC	315.340	1,015.340	491.229	723.092	13,321.247
ROC + DB	26.295	74.471	33.701	47.456	1052.457
FC	57.330	215.132	108.760	176.104	554.331
FC + DB	4.591	14.944	7.633	13.098	40.919
RankCorr	388.149	585.558	448.422	618.094	21654.163
RankCorr + DB	67.956	127.577	125.301	160.225	5398.131
sc2marker	277.122	3,150.673	368.844	240.640	17,570.998
sc2marker + DB	17.031	79.344	35.184	22.283	184.119

**Fig. 4:**
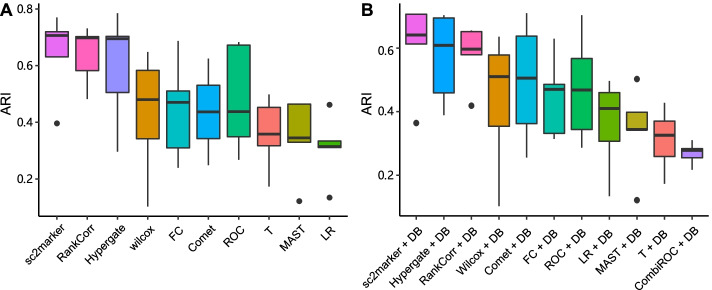
Clustering accuracy based on top 2 markers for all the evaluated methods. Clustering performance as measured by the adjusted Rand index (ARI) by considering the combination of all top two markers per method. Distribution of the ARI for top2 markers without (**A**) and with the antibody DB (**B**)

We investigated the expression patterns of the top two markers per cell of all the methods for the MCA-lung data set. As an additional benchmarking, we have performed clustering analysis by only a gene expression matrix with only the markers selected by a given method. Clustering was performed using Seurat’s Louvain algorithm with default parameters. We evaluated results by contrasting the clusters with the cell labels using the adjusted Rand index. As the use of the antibody DB reduces the marker space (and the ARI scores), we only compare methods with and without DB independently. As seen in Fig. 4, sc2marker is ranked first followed closely by RANKCORR and Hypergate. This results supports the discriminate power of small marker panels selected by sc2marker.

**Fig. 5:**
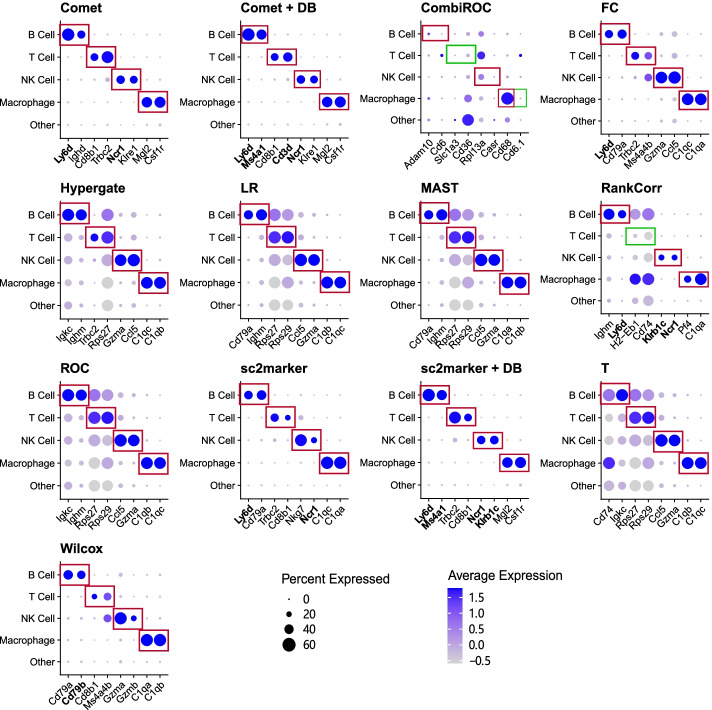
Top markers per cell type for Mouse Cell Atlas (MCA)-Lung for all the evaluate methods: Dotplots with the top two selected markers per cell type as predicted in the MCA-Lung single-cell data set. The size of the dot indicates the number of cells expressing the gene and color intensity indicates the expression level. Specific markers should be within the red boxes. Bold type indicates markers that are true labels; squares indicate cells the markers are associated with

Finally, we investigate the expression of the top two markers per cell for selected methods in the MCA-lung data set (Fig. 5). Ideally, markers should have high expression specificity; i.e., they are expressed only in the target cells. The top markers selected by sc2marker-DB and COMET-DB, the methods that performed well in ranking the MCA-lung data set (Fig. 5), had the desired cell-specific gene expression. The *t*-test, which performed worst in ranking the MCA-lung data set, selected cell markers that were expressed in other cells; i.e., Cd74, which was selected as a B cell marker, also had high expression in macrophages (Fig. 5C). Moreover, sc2marker+DB ranked two true label markers as the top two for B cells and NK cells. Similar results were observed in other data sets.

### Detection of imaging markers for rare bone marrow stromal cells

Next, we applied sc2marker and the competing methods for a characterization of mouse bone marrow with 32 cell types, including mesenchymal, immune, neuronal, endothelial, and hematopoietic progenitors cells [5]. Among others, Baccin and colleagues used the ROC method to find cell-specific markers for imaging [5]. They validated several markers for mesenchymal cell subtypes such as Cxcl12 in Adipo-CAR and Osteo-CAR cells and Alpl in Osteo-CAR cells. sc2marker also ranked these two markers: $\#$2 for Cxcl12 in Adipo-CAR and $\#$5 in Osteo-CAR, and $\#$4 for Alpl in Osteo-CAR. FC and ROC ranked Cxcl12 $\#$1 in Adipo-CAR, and ROC ranked Cxcl12 $\#$5 in Osteo-CAR cells. None of the other competing method ranked either of these two markers among the top five markers (see Additional files 7 and 8 for the complete results).

**Fig. 6:**
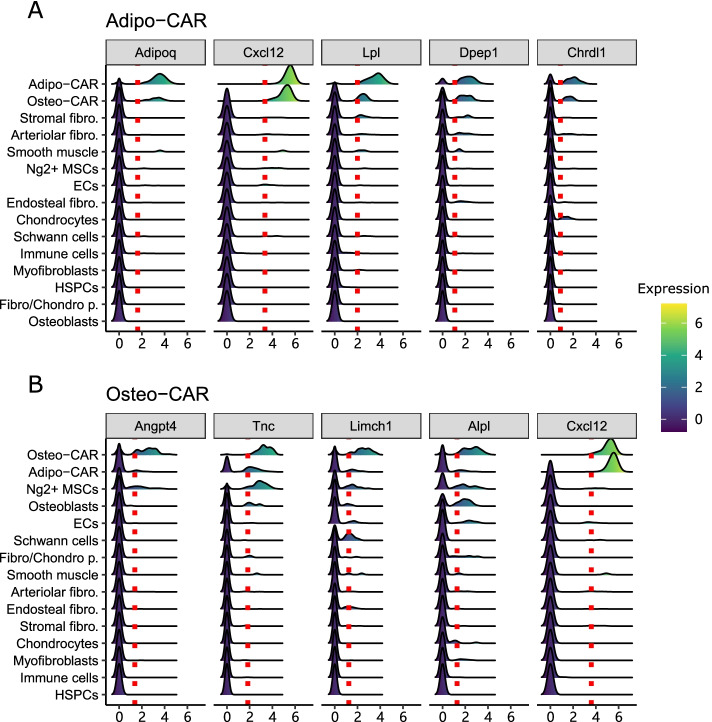
Top five markers predicted by sc2marker in Adipo-CAR and Osteo-CAR cells: Ridge plots of top five markers identified in the mouse bone marrow data set in Adipo-CAR **(A)** and Osteo-CAR **(B)** cells. Traced lines indicate the threshold detected by sc2marker. The hematopoietic, immune, and endothelial cell sub-populations are grouped for clarity

Next, we consider if sc2marker could detect markers that were more specific for Adipo-CAR and Osteo-CAR because the Cxcl12 marker reported in [5] did not differentiate between Adipo-CAR and Oste-CAR cells (Fig. 6); i.e., the expression of Cxcl12 was high in both cells (Fig. 6). We found that the markers ranked $\#$1 and $\#$3 for Adipo-CAR (Adipoq and Lpl) had more specific expression than Cxcl12 in Adipo-CAR cells (Fig. 6). sc2marker also detected two markers in Osteo-CAR (Angpt4 and Tnc) that had more specific expression than Alpl in Osteo-CAR cells.

In a recent study in Adipoq-Cre reporter mice, Adipoq+ cells were confirmed to define a population of mesenchymal cell-derived adipogenic progenitors [28]. This finding supports the potential value of Adipoq as a marker for Adipo-CAR cells. Moreover, an expression analysis of these cells indicated that they expressed Lpl, suggesting that Lpl was an alternative marker for Adipo-CAR cells. Osteo-CAR-specific markers include angiopoietin-related protein 4 (Angpt4) and tenascin-C (TnC), which are related to angiogenesis and extracellular matrix, respectively, and have been previously shown to have protein expression that is specific to bone marrow mesenchymal cells [28, 29]. Together, these results support the ability of sc2marker to find imaging markers.

**Fig. 7:**
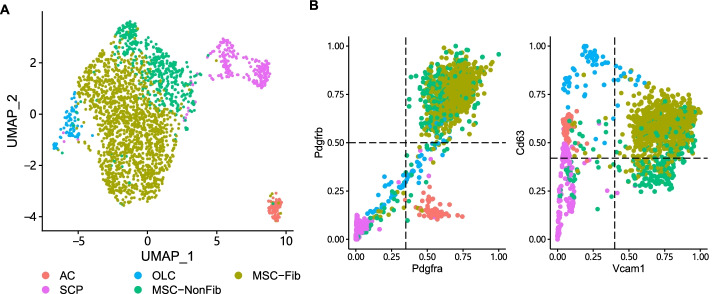
Marker detection of myelofibrosis associated MSCs UMAP demonstrating the clustering of mouse bone marrow stromal cells (**A**) and scatter plots with the expression of cells for marker genes detected by sc2open (Pdgfbra, Pdgfbrb, Cd63 and Vcam1). Expression values were imputed with MAGIC [30] (**B**)

We also evaluate sc2marker and competing methods in the characterization of bone marrow stromal cells associated with myelofibrosis in mouse [4]. There, clustering analysis revealed eight clusters: four mesenchymal stromal cells (MSCs), an osteolineage cell cluster (OLCs), an adventitial fibroblast cluster (ACs) and two clusters of schwann cell progenitors (SCPs). Pathway and cell composition analysis indicated that two of the four found MSC clusters are reprogrammed in disease and that these cells function as the key cellular drivers of fibrosis [4]. Using a FACS panel consisting of PDGFRA, PDGFRB, CD63 and VCAM1, the difference between these fibrosis-driving MSCs (MSC-Fib) and non-fibrosis driving MSCs (MSC-NonFib) could be further characterized in vitro. We used this dataset to test sc2marker and competing methods ability to find marker genes using the flow cytometry DB. Interestingly, only sc2marker returns the same four markers validated in [4]. Some of the competing methods, Hypergate, ROC and Wilcox’s test could detect three out of the four markers (Table 4). The better predictive performance of sc2marker is also confirmed by a higher ARI score after clustering cells by using the marker genes (Table 4) This is further supported by the good separation of the MSC-Fib cells from other cells by using imputed [30] expression values of these markers (Fig. 7B).

**Table 4:** Top 4 cell markers for MSC-fib in mouse stromal cells and ARI value after clustering the cells with the selected markers

Methods	Top1	Top2	Top3	Top4	ARI
sc2marker	**Vcam1**	**Pdgfrb**	**Pdgfra**	**Cd63**	**0.1882297**
MAST	Aplp1	Cldn11	**Vcam1**	Apod	0.1830788
LR	**Vcam1**	**Pdgfrb**	Aplp1	Cryab	0.1697615
Wilcox	**Vcam1**	**Pdgfrb**	Prnp	**Pdgfra**	0.1685325
ROC	**Vcam1**	**Pdgfrb**	**Pdgfra**	Prnp	0.1674651
Hypergate	**Vcam1**	**Pdgfrb**	**Pdgfra**	Lamp1	0.1491762
RankCorr	**Pdgfra**	**Pdgfrb**	Prnp	Aplp1	0.1436412
T	**Vcam1**	**Pdgfrb**	Aplp1	Cryab	0.1367963
CombiROC	Itgb4	**Vcam1**	Ly9	Il2rg	0.1296453
Comet	**Vcam1**	Pdgfrb	Il1r1	**Pdgfra**	0.1259644

Bold indicate validated markers. Methods are sorted by decreasing ARI

## Discussion

We have shown that sc2marker provides a unique framework for selecting gene markers from scRNA seq data for subsequent imaging and flow cytometry analysis. sc2marker has two major methodological advantages over competing methods: (1) it has a comprehensive antibody database for either imaging or flow cytometry analysis, and (2) its feature selection method considers the distance of the cells to the threshold value (or margin) and favors thresholds that maximize the distance to true positive and true negative predictions.

Our benchmarking analysis on five scRNA-seq data sets showed the advantage of sc2marker over competing methods. Using sc2marker without an antibody database showed that sc2marker performed better (lower rankings and best clustering results) than the other methods in the majority of evaluated data sets. A comparative analysis of the expression patterns of the top ranked markers indicated that sc2marker detected markers that were specifically expressed in the target cells, whereas the common statistical approaches used in Seurat tended to select markers that were also expressed in other cell types related to the target cell type. Another methodological advantage is the introduction of a comprehensive antibody data base. Its use reduced the computational time and ranking scores for all evaluated methods. Our analysis of imaging markers and flow cytometry for rare stromal cells further highlighted the advantage of sc2marker over competing methods.

sc2marker is implemented in an easy to use R library that can be used with scRNA-seq data analyzed by Seurat [3]. sc2marker provides graphical and tabular reports that include annotation of the selected markers; i.e., source of the antibody annotation and link to the antibody register. Furthermore, sc2marker can be expanded with a user provided antibody database that can be used in combination with existing ones. These features provide a simple and direct way for users to derive new markers from single-cell data by combing the known expertise of the laboratory.

The relationship between the amount of RNA transcripts, as measured by RNA-seq, and the abundance level of protein, as measured in CITE-seq, imaging and flow cytometry, is not one to one. A good example is the discordance of the expression values of the PTPRC gene and its two protein isoforms CD45RA and CD45RO (Additional file 9: Fig. S1). Another example is CD4, which is a well established marker for some T cell subsets. For the CITE-seq human-BM and human-PBMC data sets, CD4 has a expression specific to T cells at the protein level (Additional file 9: Figs. S2, S3). However, at the RNA level, CD4 has higher expression in monocytes than in T cells . This is a clear limitation of any scRNA-seq based marker detection algorithm, which should be considered by its users.

Also, sc2marker (and any competing method) assumes that all the negative cells are present in the scRNA-seq data. Therefore, to select imaging markers, users should use scRNA-seq data that were obtained from all major cell types of a given tissue. This is the case for the stromal cell scRNA-seq data set, which contains data for all major stromal and hematopoietic cells in the bone marrow. To identify flow cytometry markers, scRNA-seq data of pre-sorted cell populations can be used; however, the newly detected markers need to be combined with the flow scheme that was used before the scRNA-seq experiment was conducted. An interesting feature of some competing methods, e.g. COMET, RANKCORR and CombiROC, is the fact that they are able to find marker panels combining a few markers. It comes, however, with an exponential increase in the computational requirements. The extension of sc2marker to consider marker combinations by exploring efficient max-margin classifiers is an interesting path for future work.

## Supplementary Information

**Additional file 1:** Results of markers detection for MCA-Spleen dataset with sc2marker and other competing mehtods.

**Additional file 2:** Results of markers detection for MCA-Lung dataset with sc2marker and other competing methods.

**Additional file 3:** Results of markers detection for HCA-Lung&PBMC dataset with sc2marker and other competing methods.

**Additional file 4:** Results of markers detection for HCA-BM dataset with sc2marker and other competing methods.

**Additional file 5:** Results of markers detection for HCA-PBMC dataset with sc2marker and other competing methods.

**Additional file 6:** Rankings of true markers for MCA-Spleen, MCA-Lung, HCA-Lung&PBMC, HCA-BM and HCA-PBMC datasets.

**Additional file 7:** Results of markers detection for Osteo-car cell populations in stromal cells with sc2marker and other competing methods.

**Additional file 8:** Results of markers detection for Adipo-car cell populations in stromal cells with sc2marker and other competing methods.

**Additional file 9:** Supplementary Figures - Detection of cell markers from single cell RNA-seq with sc2marker.

## Data Availability

sc2marker is available as a R open source package in github (https://github.com/CostaLab/sc2marker). This includes tutorials and scripts used for analysis and all data sets presented in this manuscript. The scRNA-seq of stromal cells analysed during the current study are available in the zenodo repository https://doi.org/10.5281/zenodo.3979087.

## Abbreviations

AARI: Adjusted Rand Index
FC: Fold change
FNR: False positive rate
IHC: Immunohistochemistry
ICC: Immunocytochemistry
MCA: Mouse Cell Atlas
MSC: Mesenchymal stromal cells
NK: Cell Natural killer cells
PBMC: Peripheral blood mononuclear cell
ROC: Teceiver operating characteristic
scRNA-seq: Single-cell RNA sequencing
TPR: True positive rate
UMAP: Uniform manifold approximation and projection
WNN: Weighted Nearest Neighbor Analysis
