# Supplementary material for: Detection of cell markers from single cell RNA-seq with sc2marker
Source: BMC Bioinformatics. 2022 Jul 12;23:276. doi: 10.1186/s12859-022-04817-5 (PMC9281170; doi:10.1186/s12859-022-04817-5)
Supplement: Supplementary file 9 — Additional file 9: Supplementary Figures - Detection of cell markers from single cell RNA-seq with sc2marker. [file 12859_2022_4817_MOESM9_ESM.pdf]

Supplementary Figures: Detection of cell markers from single cell RNA-seq with sc2marker

May 2022

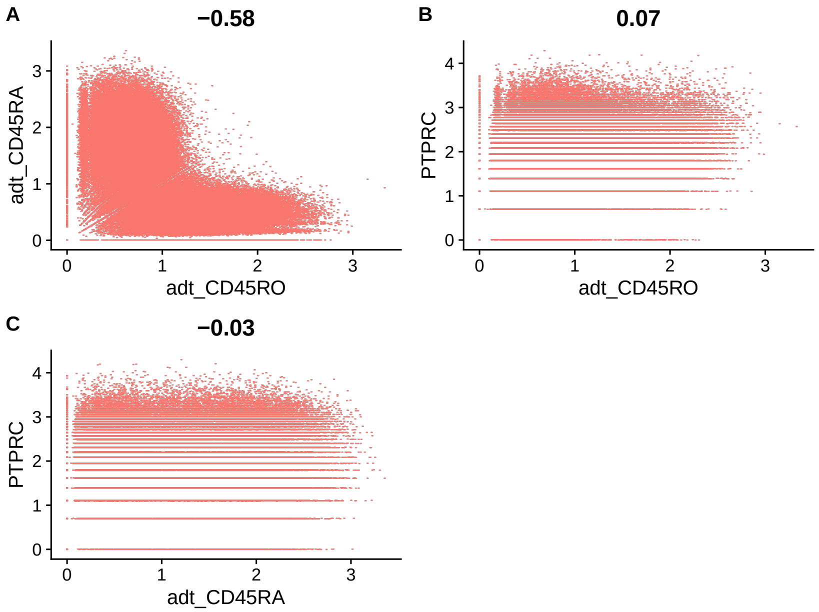

Figure 1: **Expression of CD45RA, CD45RO in Human-PBMC data.** Scatter plot of the gene PTPRC and the abundances of its two protein isoforms in Human-PBMC dataset: **(A)** CD45RA vs. CD45RO, **(B)** PTPRC vs. CD45RO, **(C)** PTPRC vs. CD45RA.

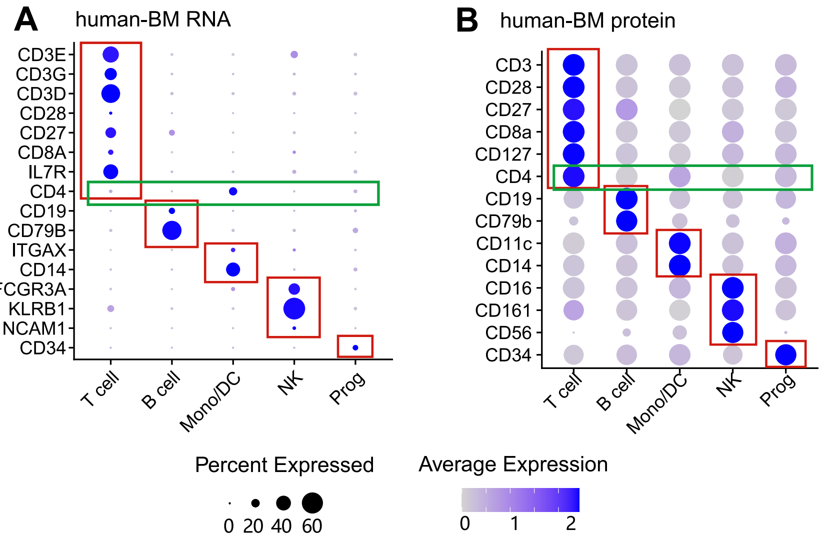

Figure 2: **Dot plot of RNA and protein modality of human-BM CITE-seq data.** Dot plot of markers used in Human-PBMC dataset (**(A)** RNA, **(B)** protein). We highlight the expression of CD4, which is a good marker for CD4 T cells in protein space, but not at RNA space.

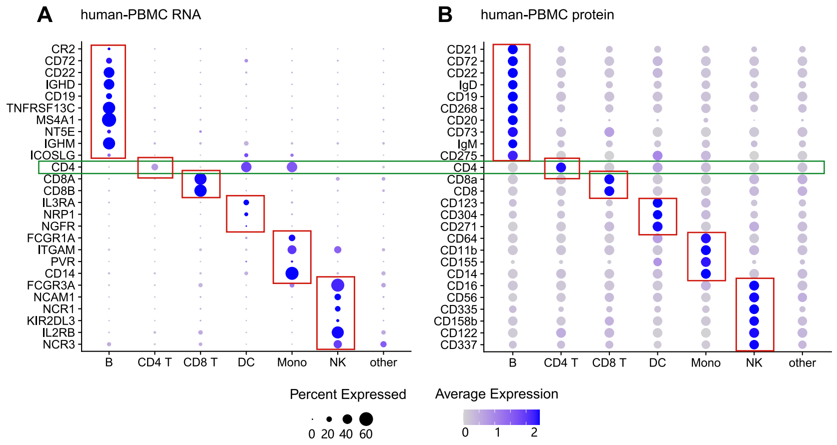

Figure 3: **Dot plot of RNA and protein modality of human-PBMC CITE-seq data.** Dot plot of markers used in Human-PBMC dataset. **(A)** RNA, **(B)** protein. We highlight the expression of CD4, which is a good marker for CD4 T cells in protein space, but not at RNA space.
